# Injectable Silk Fibroin–Puerarin Hydrogels with Tunable Supramolecular Organization as a Potential Platform for Tissue Engineering

**DOI:** 10.1021/acsomega.6c02412

**Published:** 2026-05-13

**Authors:** Bruna V. Quevedo, Bianca Sabino Leocádio Antunes, Saeed Safari, David Hubbard, Daniel Komatsu, Menekse Ermis, Eliana Aparecida de Rezende Duek

**Affiliations:** † Postgraduate Program in Materials Science (PPGCM), Federal University of São Carlos (UFSCar), Sorocaba, SP 18052-780, Brazil; ‡ Laboratory of Biomaterials, Faculty of Medical Sciences and Health (FCMS), Pontifical Catholic University of São Paulo (PUC-SP), Sorocaba, SP 18030-070, Brazil; § 678581 Terasaki Institute for Biomedical Innovation (TIBI), Los Angeles, California 91367, United States

## Abstract

Injectable supramolecular hydrogels represent an emerging class of biomaterials with significant potential for minimally invasive tissue engineering and drug delivery applications. In this study, the effects of varying puerarin (PUE, 1–5%) contents, a bioactive isoflavonoid capable of gelation, on the physicochemical and in vitro biological properties of silk fibroin (SF) were systematically evaluated. Hydrogel formation was driven by hydrogen-bonding interactions between SF and PUE, as confirmed by FTIR analysis, without altering the crystalline structure of SF, as evidenced by X-ray diffraction. Microstructural analysis by scanning electron microscopy (SEM) revealed that increasing PUE content progressively reduced pore size and generated a denser polymeric network, resulting in a decrease in swelling capacity. Thermal analyses (TGA/DSC) demonstrated the combined thermal stability and degradation behavior of the resulting system. Rheological characterization showed a marked increase in viscosity and a predominance of storage modulus (*G*′) over loss modulus (*G*″), indicating the formation of a mechanically stable supramolecular network with tunable viscoelastic properties and injectability. In vitro biological assays demonstrated that the hydrogels are cytocompatible with HDF cells. Moreover, in vitro scratch assays demonstrated cell migration and complete wound closure within 72 h. In summary, the results demonstrate that PUE incorporation modulates the supramolecular organization and physicochemical properties of SF hydrogels in a concentration-dependent manner, enabling tunable and cytocompatible injectable systems with potential relevance for soft tissue biomaterial applications.

## Graphical abstract

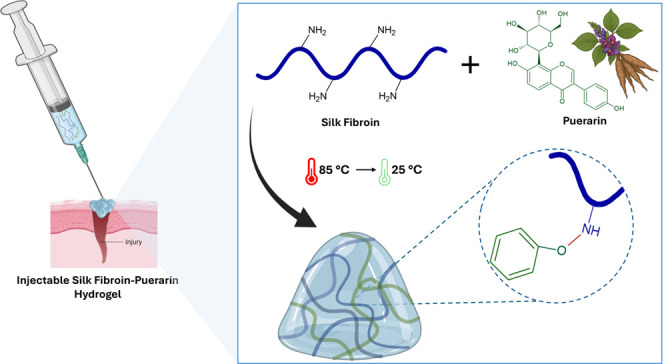

## 1 Introduction

Biomaterials engineering has played a pivotal role in advancing regenerative therapies, particularly through the development of injectable systems capable of adapting to complex biological microenvironments and promoting tissue regeneration in a minimally invasive manner.^1^ Among various biomaterials, hydrogels stand out due to their three-dimensional structure resembling the extracellular matrix, high water retention capacity, softness, flexibility, porosity, permeability, and excellent biocompatibility, making them highly promising for soft tissue regeneration and controlled drug-delivery applications.^2^ Injectable hydrogels have gained prominence in the biomedical field owing to their ability to be locally and precisely administered into anatomically challenging regions or tissue interfaces undergoing difficult healing processes.^3^ These systems can be engineered to undergo in situ gelation or to respond to mechanical stimuli, such as shear-thinning behavior, via cross-linked polymeric networks, thereby expanding their potential for use in advanced clinical applications.^4^

Hydrogels can be formulated from synthetic or natural materials, whose properties directly affect their performance in biomedical applications. Synthetic polymers, such as poly­(lactic acid) (PLA), polyethylene glycol (PEG), and poly­(glycolic acid) (PGA), are widely used due to their processability, mechanical stability, and reproducibility. However, these materials often exhibit low bioactivity, lack specific biological signals, and, in some cases, produce acidic byproducts during degradation.^5^ On the other hand, natural polymers such as collagen, gelatin, and silk fibroin offer biocompatibility, biodegradability, and ability to promote favorable cellular interaction. Nonetheless, they tend to have limitations in mechanical stability and in the precise control of their physicochemical properties.^5,6^ In this context, strategies based on chemical and/or physical cross-linking have proven effective in overcoming the individual limitations of different materials used in hydrogel formulations. Chemically cross-linked hydrogels are formed through permanent covalent bonds, which provide structural stability but may involve complex and costly reactions, as well as the use of potentially toxic cross-linking agents. Conversely, physically cross-linked hydrogels overcome these drawbacks by being stabilized through noncovalent interactions, including hydrogen bonding, hydrophobic interactions, electrostatic attractions, and ionic interactions.^7^

Among naturally derived biopolymers, silk fibroin (SF), obtained from the cocoon of the silkworm *Bombyx mori*, has attracted considerable attention as a hydrogel-forming matrix. This interest arises from its favorable biological performance, including excellent biocompatibility and biodegradability, as well as its adjustable mechanical characteristics and capacity to promote cell adhesion, proliferation, and differentiation.^8^ SF is composed of long polypeptide chains of approximately 390 kDa and shorter chains of around 26 kDa, which are linked via disulfide bonds. The long-chain fraction of SF consists of repetitive sequences of hydrophobic amino acids, such as glycine, alanine, and serine. This domain adopts a crystalline β-sheet conformation, which imparts significant mechanical strength to the material. In contrast, the short-chain fraction, corresponding to the hydrophilic domain, is primarily composed of disordered, amorphous amino acids that assume an α-helical conformation, providing elasticity and toughness to the SF network.^9^

Despite its intrinsic properties, the SF hydrogel exhibits poor mechanical strength, making it susceptible to mechanical fragility under stress. This limitation can be overcome by inducing the formation of β-sheet structures, which confer greater rigidity to the system.^10^ Such structural transitions can be achieved through various techniques, including enzymatic, chemical, or physical cross-linking. Among these strategies, physical cross-linking based on supramolecular hydrogels composed of natural low-molecular-weight compounds has gained increasing attention due to their intrinsic inherent biocompatibility, potential pharmacological activity, natural abundance, and ability to form reversible networks in response to external stimuli, while requiring low gelator concentrations and allowing facile chemical modifications.^11^

In this regard, puerarin (PUE) has emerged as promising yet relatively underexplored self-gel material. Chemically identified as [7-hydroxy-3-(4-hydroxyphenyl)-1-benzopyran-4-one-8-(β-d-glucopyranoside)], PUE is a naturally occurring bioactive flavonoid isolated from the roots of *Pueraria lobata*, commonly known as kudzu root.^12^ PUE can form gels upon contact with water, resulting in supramolecular hydrogels through a heating and subsequent cooling process.^13^ Furthermore, PUE can act as a low-molecular-weight gelling agent in the formation of supramolecular hydrogels. The plant contains a wide variety of phytochemicals, such as genistein and daidzein, with PUE being the primary agent responsible for its pharmacological effects.^14^ PUE exhibits remarkable antioxidant, anti-inflammatory, antiapoptotic, pro-angiogenic, and antihyperglycemic activities.^15,16^ Moreover, PUE demonstrates extensive pharmacological effects relevant to the treatment of cerebrovascular and cardiovascular disorders, such as angina, myocardial infarction, heart failure, and viral myocarditis.^17^ According to Yuan et al.,^18^ PUE can be incorporated into hydrogel matrices to form networks with interpenetrating characteristics and bioactive properties. However, its use as a structural reinforcement component in polymeric matrices has been rarely reported.

Cai et al.^19^ demonstrated that the supramolecular PUE hydrogel exhibits remarkable antioxidant properties and maintains high stability in acidic environments. More recently, Luo et al.^20^ highlighted the potential of PUE-based hydrogels as controlled release systems, capable of delivering the chemokine SDF-1, which promotes bone marrow stem cell recruitment and macrophage polarization, ultimately supporting tissue regeneration. In contrast, although Yang et al.^21^ developed an injectable hydrogel composed of SF and PUE, their study focused on the incorporation of gallium ions and on evaluation of its antibacterial and hemostatic properties for application in the treatment of infected wounds.

Although previous studies have demonstrated the potential of PUE in hydrogel-based systems, investigations addressing the role of PUE concentration in modulating the supramolecular organization and structural properties of SF hydrogels obtained exclusively through physical cross-linking remain limited. The incorporation of PUE into SF matrices may act as a molecular modulator capable of influencing the self-organization of polymer chains via noncovalent interactions, including hydrogen bonding and π–π stacking. These interactions contribute to the development of more organized and tunable structures. However, the correlation between PUE concentration and the structural, mechanical, and injectable properties of these systems remains insufficiently explored.

Therefore, the present study investigates injectable supramolecular SF hydrogels containing different PUE concentrations (1–5%), aiming to elucidate the role of PUE as a structural modulator in physically cross-linked networks. In this context, a systematic correlation is established between PUE content and the supramolecular organization, microstructural features, viscoelastic behavior, and biological performance of the system. In contrast to previously reported approaches, which often rely on combined self-assembly mechanisms or additional cross-linking strategies, the present work is based exclusively on intrinsic physical interactions between SF and PUE, enabling a direct assessment of the influence of PUE on network formation and stability in aqueous environments.

The results demonstrate that increasing PUE content promotes the transition from a noncohesive SF solution to a structurally stable and extrudable hydrogel, accompanied by enhanced network compaction, modulated swelling behavior, and tunable viscoelastic properties. Moreover, the systems maintain high cytocompatibility, with the potential to stimulate metabolic activity and cell migration. This coupling between mechanical reinforcement, injectability, and biological response represents a level of functional integration that is typically not achieved in physically cross-linked SF systems without the incorporation of cross-linking agents or additives. Collectively, these findings demonstrate that modulation of PUE content enables the simultaneous tuning of mechanical and biological properties, highlighting the functional versatility of the system and its potential for applications in soft tissue engineering and minimally invasive therapies.

## 2 Materials and Methods

### 2.1 Materials

Silk fibroin (SF) obtained from silkworm cocoons of the *Bombyx mori* species was kindly provided by the Laboratory of Applied Biochemistry at Lund University (Sweden). Calcium chloride dihydrate (CaCl_2_·2H_2_O, 147.02 g/mol) was purchased from Synth (Brazil), and sodium carbonate (Na_2_CO_3_, 105.99 g/mol) was acquired from Êxodo Científica Ltd. (Brazil). Ethanol 99.5% (C_2_H_6_O) was obtained from Dinâmica Contemporânea Ltd. (Brazil). Cellulose dialysis membranes with a diameter of 76 mm were purchased from Sigma-Aldrich (USA). Puerarin (PUE) (purity >99%) root powder (*Pueraria lobata*) was obtained from Zhongxiang Shanling Ltd. (China).

### 2.2 Methods

#### 2.2.1 Extraction and Preparation of Silk Fibroin

The SF fibers were subjected to a degumming process according to adaptations of the methodology described by Araújo et al.^22^ Initially, the SF fibers were cut and immersed in a Na_2_CO_3_ solution (0.5% w/w). The solution was transferred to an autoclave and subjected to thermal treatment at 120 °C for 15 min. Following this step, the degummed SF was rinsed three times with distilled water to eliminate residual sericin and Na_2_CO_3_. The SF was dried in an oven at 50 °C for 24 h. Subsequently, the dried SF was solubilized in a ternary solvent system composed of CaCl_2_/ethanol/H_2_O (molar ratio of 1:2:6) at 85 °C until complete dissolution (approximately 10 min). The resulting SF solution was dialyzed using a cellulose membrane with ultrapure water as the dialysis medium for 72 h. After dialysis, the solution was centrifuged at 5000 rpm and 4 °C for 15 min, and this step was repeated until no visible precipitate remaining. The purified solution was then lyophilized at −85 °C for 4 days to ensure complete drying, and the obtained SF was stored in a desiccator until use. A fraction of the dialyzed solution was transferred to Petri dishes and covered with perforated plastic film to allow controlled solvent evaporation at room temperature (∼25 °C). After approximately 3 weeks, spontaneous formation of the silk fibroin gel (SF-G) was observed, resulting from the self-assembly of the protein chains. The remaining SF solution was stored under refrigeration (4 °C) prior to subsequent characterization.

#### 2.2.2 Preparation of Hydrogel

SF and PUE-based hydrogels were prepared following a modified version of the method described by Pang et al.^13^ SF solutions (4% w/v) were prepared by dissolving lyophilized SF in deionized water under stirring for 5 min. Subsequently, PUE was incorporated into SF solutions at concentrations of 1, 2, 3, 4, and 5% w/v. The mixtures were stirred at 200 rpm for approximately 5 min to promote uniform dispersion and complete homogenization of PUE within the polymeric solution. The resulting mixtures were then heated to 85 °C under continuous stirring until 5 min. After this step, the systems were allowed to cool to room temperature (25 °C) to ensure complete formation of the supramolecular hydrogels (Figure S1). The formulations used for hydrogel preparation are summarized in Table 1.

**Table 1:** Hydrogel Formulations in % w/v Concentrations

**sample**	**SF (%)**	**PUE (%)**
SF	4	
SF + 1% PUE	4	1
SF + 2% PUE	4	2
SF + 3% PUE	4	3
SF + 4% PUE	4	4
SF + 5% PUE	4	5

### 2.3 Characterization

#### 2.3.1 Fourier Transform Infrared Spectroscopy (FTIR)

The FTIR spectra was acquired using an ATR accessory coupled to a Spectrum 65 spectrophotometer (PerkinElmer, USA). Measurements were over the 4000–500 cm^–1^ range, with a spectra resolution of 4 cm^–1^ and 32 accumulated scans.

#### 2.3.2 X-ray Diffraction (XRD)

Diffraction patterns of PUE powder samples, lyophilized SF samples, and SF+PUE hydrogels (1–5%) were analyzed over a 2θ diffraction angle range of 5 to 45°, using a scanning rate of 2°/min with an XRD instrument (Shimadzu 6100). The diffraction measurements were carried out using Cu Kα radiation (λ = 1.5406 Å), with the X-ray source operate at 40 kV and a current of 30 mA.

#### 2.3.3 Scanning Electron Microscope (SEM)

Lyophilized SF and SF+PUE hydrogels (1–5%) were previously cryo-fractured in liquid nitrogen. The fracture specimens were then coated with a thin gold layer using a sputter coater (EM ACE200, Leica, Germany). Morphological characterization was carried out by scanning electron microscope (SEM) using a ZEISS EVO MA15 instrument operated at an accelerating voltage of 3 kV. Pore size distribution was determined from the micrographs with ImageJ software, considering approximately 50 pores for statistical analysis.

#### 2.3.4 Swelling Test

The water uptake capacity of the samples was evaluated through an apparent swelling assay using previously prepared SF and SF+PUE hydrogels. The samples were initially lyophilized and weighed, then transferred to 15 mL Falcon tubes. Subsequently, the samples were transferred to 10 mL of phosphate-buffered saline (PBS, pH 7.2) and maintained at 37 °C under incubations conditions. Due to the high-water absorption capacity and the resulting macroscopic changes of the samples, they were maintained within the tubes throughout the entire experiment, avoiding direct handling. At predetermined time intervals, the medium was carefully removed, and the system (tube containing the hydrated sample) was weighed to determine the weight at time *t*, after subtracting the weight of the empty tube. After weighing, the medium was reintroduced into the tube to continue the experiment. The assay was conducted for a total period of 432 h. The swelling assay was conducted in triplicate, and the swelling ratio (%) was determined using the initial dry weight of the samples (*W*_0_) and the weight measured after hydration at each time interval (*W_t_*), as described in eq 1.

$$\mathrm{Swelling}(\%)=\left[\frac{W_{t}-W_{0}}{W_{0}}\right]\times 100\%$$ 1

#### 2.3.5 In Vitro Degradation Analysis

The initial dry weight of lyophilized SF and SF+PUE hydrogels (1–5%) was accurately measured. The samples were subsequently immersed in phosphate-buffered saline (PBS, pH 7.2) and maintained at 37 °C. At predetermined time points, the hydrogels were collected, lyophilized, and weighed to determine the degradation rate. All experiments were performed in triplicate. The weight loss percentage (*W*_L_) was determined according to eq 2:

$$W_{L}(\%)=\left[\frac{W_{0}-W_{1}}{W_{0}}\right]\times 100\%$$ 2

Where *W*_0_ corresponds to the initial dry weight of the hydrogel before exposure do hydrolytic degradation conditions, and *W*_1_ refers to the dry weight obtained after degradation.

#### 2.3.6 Thermal Analysis

Thermogravimetric analysis (TGA) was carried out on PUE powder and lyophilized hydrogel samples using a Discovery TGA 55 instrument (TA Instruments, USA). The measurements were performed with a temperature range from 25 to 500 °C under a nitrogen atmosphere with a flow of 40 mL/min, applying a heating rate of 10 °C/min. Differential scanning calorimetry (DSC) analysis were conducted using a Discovery DSC 25 system (TA Instruments, USA), under a dynamic nitrogen flow of 250 mL/min. The samples were first heated from 25 to 200 °C, then cooled to −50 °C with a 1 min isotherm step, followed by a second heating cycle up to 350 °C. All heating and cooling stages were performed at a rate of 10 °C/min.

#### 2.3.7 Rheological Property of Hydrogels

The rheological behavior of the SF solution and the SF+PUE hydrogels (1–5%) were characterized using a DHR-2 rheometer (TA Instruments, USA). Measurements were performed at 37 °C using a cone–plate configuration (40 mm diameter, 0.55 mm gap). The viscosity profile was obtained as a function of shear rate over the range of 0.001 to 100 s^–1^. The mechanical strength of the hydrogels was assessed through dynamic oscillatory tests, specifically amplitude sweep tests, of the *G*′ and *G*″ moduli as a function of the % deformation (0.01–100%), at a constant frequency value (1 Hz), which were used to identify the Linear Viscoelastic Region (LVR) and the yield point (solid-like to liquid-like). Frequency sweep measurements were carried out over a range of 0.1 to 10 Hz to evaluate the storage modulus (*G*′) and loss modulus (*G*″). The LVR value for all samples was 1%. The sample was equilibrated at 37 °C for 1 min prior to measurement for thermal stabilization. To prevent evaporation during the assay, a mineral oil ring was applied over the sample. The gelation kinetics were evaluated by time sweep analysis. The hydrogel precursors were initially heated to 80 °C and subsequently transferred to rheological evaluation at a fixed temperature of 37 °C. The time-dependent evolution of the *G*′ and loss *G*″ was continuously monitored.

#### 2.3.8 Injectability Test

Injectability analyses of the samples were performed using a TAXTPlus Texture Analyzer (Stable Micro Systems, UK), previously calibrated with a 5 kg load cell, in compression mode. The syringe corresponding to each sample was positioned vertically in a suitable apparatus, and a beaker was used to collect the extruded sample. A 10 mm diameter analytical probe was compressed onto the plunger surface at a speed of 1 mm s^–1^ with a displacement of 20 mm. All experiments were performed in triplicate. The maximum force and dynamic sliding force, defined as the force required to sustain plunger movement during sample extrusion, were quantified using Texture Expert software. A 27G needle was used. The injectability assays were conducted at room temperature (25 ± 2 °C).

#### 2.3.9 In Vitro Biological Characterization

##### 2.3.9.1 Cytocompatibility of Hydrogels

The in vitro cytotoxicity of the hydrogels was assessed using PrestoBlue (Fisher Scientific, A13262) and Live/Dead assays (Fisher Scientific, USA), with human dermal fibroblasts (HDF) as the cellular model. HDF cells were maintained in Dulbecco’s Modified Eagle Medium (DMEM, high glucose, Gibco, USA) supplemented with 10% (v/v) fetal bovine serum (FBS, Gibco, USA) and 1% (v/v) penicillin/streptomycin (Gibco, USA). Cell cultures were incubated at 37 °C under a humidified atmosphere containing 5% CO_2_.

HDF cells were seeded into 6-well plates at a density of 1 × 10^4^ cells per well and incubated for 2 h under standard culture conditions to promote cell adhesion. Subsequently, 200 μL of SF solution and SF+PUE hydrogels, previously prepared under aseptic conditions and sterilized by UV irradiation (λ = 254 nm) for 2 h, were transferred to the upper compartment of transwell inserts equipped with polyethylene terephthalate (PET) membranes (0.4 μm pore size; Greiner Bio-One, USA), thereby establishing an indirect contact setup. Negative control (CT-) consisted of cells cultured in the absence of materials. The cultures were maintained in 5 mL of complete DMEM, under standard incubation conditions (37 °C, 5% CO_2_) for up to 6 days. Cell viability and metabolic activity were evaluated on days 1, 4, and 6 using the PrestoBlue assay, following the protocol provided by the manufacturer. Fluorescence was recorded at an excitation wavelength of 560 nm and an emission wavelength of 590 nm using a microplate reader (Varioskan LUX, Thermo Fisher). The data are presented as mean ± standard deviation from three independent experiments.

Cell viability was further evaluated using Live/Dead staining at the same time points. Fluorescence images were acquired using a BZ-X710 fluorescence microscope (KEYENCE, Japan). Calcein-AM (green fluorescence) was used to identify viable cells, while ethidium homodimer-1 (red fluorescence) indicated nonviable cells. For quantitative analysis, ten nonoverlapping fields per sample (10× magnification) were analyzed using ImageJ-Fiji software (NIH, USA). Experiments were conducted in triplicate, and cell viability (%) was determined by calculating the proportion of viable cells to the total cell population.

##### 2.3.9.2 Cytochemical Evaluation

The cytochemical assay was performed in accordance with the international standard ISO 10993–5 (2009). Vero cells, am immortalized epithelial cell line derived from the kidney of the African green monkey (*Cercopithecus aethiops*), were used in this study. The cells acquire from the Rio de Janeiro Cell Bank, Brazil (code 0245), and maintained in DMEM supplemented with 10% fetal bovine serum (FBS; Cultilab, Brazil). Cell cultures were incubated at 37 °C under a humidified atmosphere with 5% CO_2_. Cells were expanded in 75 cm^2^ culture flasks until reaching confluence. Subsequently, they were harvested and seeded into 24-well plates at a density of 1 × 10^5^ cells per well, using a final volume of 0.5 mL.

The cells were then exposed to extracts obtained from different materials, including SF and SF-based hydrogels containing 1–5% PUE, and incubated for 24 and 72 h at 37 °C under a humidified atmosphere with 5% CO_2_. For extract preparation, approximately 0.1 g of each hydrogel was immersed in 1 mL of complete DMEM and incubated at 37 °C for 24 h. Following incubation, the extracts were collected and directly used, without dilution (100%), in subsequent in vitro biological assays.^23^ Negative control (CT-) consisted of cells cultured in the absence of any material. After the incubation step, the samples were fixed, rinsed, and stained using 0.5 mL of crystal violet (CV), xylidine ponceau (XP, pH 2.5), and toluidine blue (TB, pH 4.0).^24^ Bright-field images were acquired using an optical microscope.

##### 2.3.9.3 Scratch Assay

The scratch assay was performed following a previously reported protocol.^25^ Vero cells were seeded in 24-well plates at a density of 1 × 10^5^ cells per well using complete DMEM medium. After reaching full confluence, a linear wound was created across the cell monolayer using a sterile 200 μL pipet tip. Sterile extracts of SF and SF-based hydrogels containing different concentrations of PUE (1–5%), previously prepared in DMEM medium, were used. As a negative control (CT-), cells were maintained under identical conditions without hydrogel treatment. Wound healing was evaluated by monitoring cell migration using a bright-field microscope at 0, 24, 48, and 72 h of cell culture. The wound width was quantified using ImageJ, and the percentage of wound closure was calculated according to eq 3.

$$\mathrm{Wound}\;\mathrm{Closure}(\%)=\left[\frac{\left(At_{0\;h}-At_{\Delta h}\right)}{At_{0\;h}}\right]\times 100\%$$ 3

Where *At*_0 h_ represents the wound area measured immediately after scratching (*t* = 0), and *At*_Δ*h*_ corresponds to the wound area determined at 24, 48, and 72 h post scratch.

#### 2.3.10 Statistical Analysis

Statistical comparisons among different time points were performed using one-way ANOVA, followed by Tukey’s post hoc test. A 95% confidence level was adopted, and statistical significance was defined as *p* < 0.05 (*), *p* < 0.01 (**), *p* < 0.001 (***), and *p* < 0.0001 (****).

## 3 Results and Discussion

### 3.1 Synthesis and Characterization of Supramolecular Hydrogel

The schematic illustration (Figure 1A) depicts the proposed supramolecular self-assembly mechanism between SF and PUE during the thermally induced sol–gel transition following the heating and cooling process. During heating, SF chains and PUE molecules are molecularly dispersed in the aqueous medium. Upon cooling, noncovalent interactions are established between the components. In this context, the hydroxyl (−OH) groups of PUE are able to interact with the amide (−NH) groups of SF through hydrogen bonding, acting as physical cross-linking points that stabilize the three-dimensional hydrogel network and enable its injectability.

**Figure 1:**
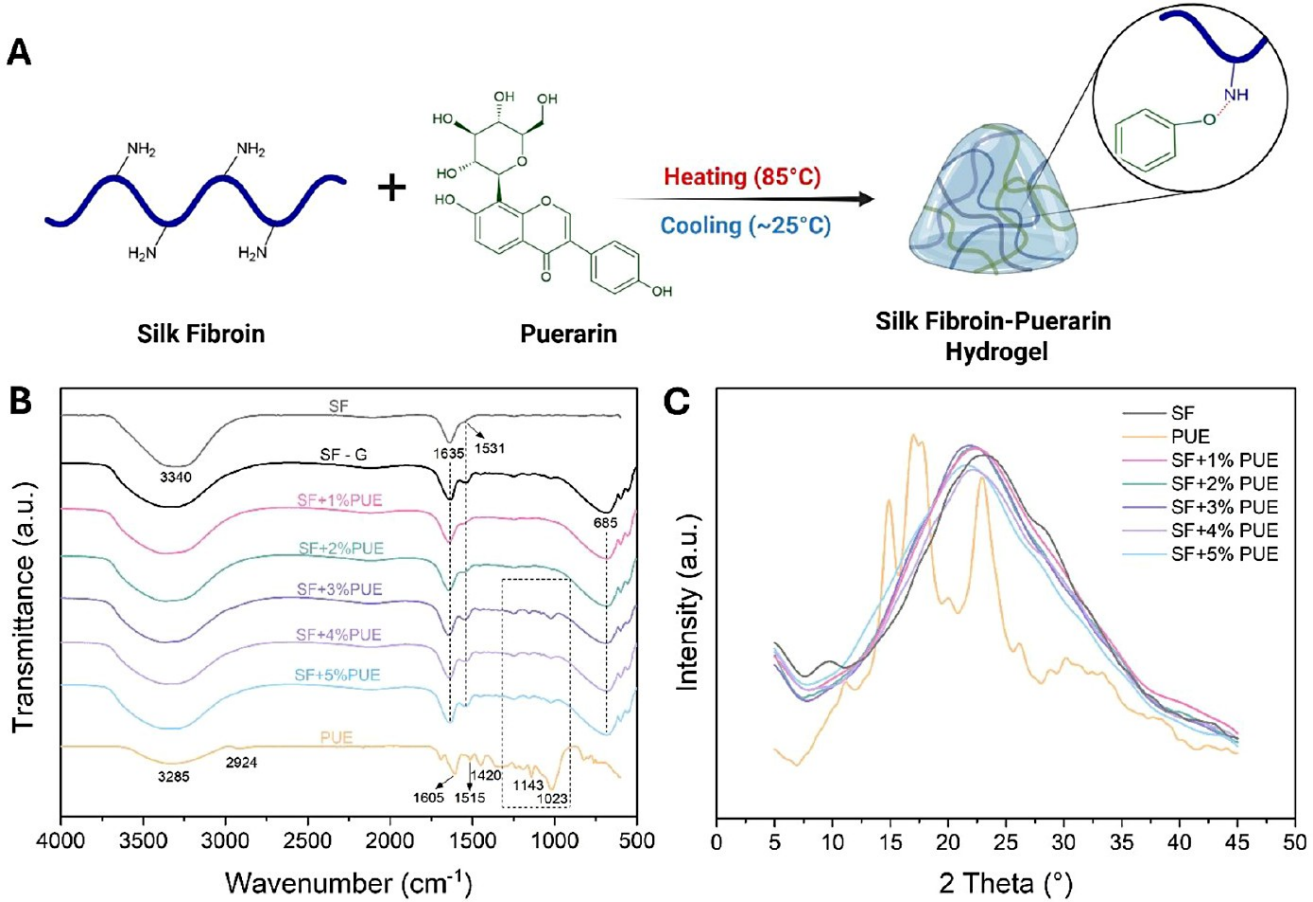
Structural characterization. (A) Schematic representation of hydrogel formation through physical cross-linking and self-assembly of silk fibroin (SF) and puerarin (PUE). (B) FTIR spectra of silk fibroin (SF) solution, SF gel (SF-G), PUE, and SF hydrogels with different concentrations of PUE (1–5%). (C) X-ray diffraction (XRD) patterns of SF, PUE, and lyophilized SF+PUE hydrogels.

Figure 1B shows the comparison of the FTIR spectra obtained for the SF, SF in gel form (SF-G), previously obtained according to a well-established gelation protocol (Section 2.2.1), and SF hydrogels containing different concentrations of puerarin (PUE) (1–5%). The infrared spectra of SF and SF-G showed a broad peak at 3290 cm^–1^, attributed to O–H stretching vibrations, overlapping with the N–H stretching of amide A groups.^26^ Characteristic peaks associated with C=O stretching of amide I were observed at 1635 cm^–1^, while the shoulder at 1531 cm^–1^ was associated with N–H bending of amide II. Both bands were associated with the random coil conformation typically found in SF.^27,28^ However, the SF-G spectrum also showed an increased intensity of the amide II band (1531 cm^–1^), indicating an enhancement in the β-sheet crystalline structure resulting from the gelation process.^29^ Additionally, the emergence of a new peak at 685 cm^–1^ was designed to amide IV, characteristic of the silk II conformation.^30^

The FTIR spectrum of PUE exhibited a broad absorption band centered at approximately 3285 cm^–1^, attributed to O–H stretching vibration characteristic of alcohols and phenolic groups, indicating the presence of hydroxyl groups in the molecular structure. The peak observed at 2924 cm^–1^ was assigned to the asymmetric stretching of C–H bonds. Additionally, the peaks located at 1605, 1515, and 1420 cm^–1^ were associated with the characteristic vibrations of the aromatic rings in PUE, confirming its phenolic structure. The peaks at 1143 and 1023 cm^–1^ were attributed to the stretching vibration of the =C–O–C linkage present in the glucopyranose unit.^31^

The infrared spectra of the SF+PUE hydrogels (1–5%) displayed the main characteristic absorption peaks of SF at 1635 and 1531 cm^–1^, corresponding to amide I and amide II, respectively. The presence of PUE was observed only in the samples with higher concentrations of the compound (SF+3%PUE, SF+4%PUE, and SF+5%PUE). The gradual incorporation of PUE into SF (SF+PUE) resulted in an increase in the intensity of the peak located at 1531 cm^–1^. This phenomenon was corroborated through the deconvolution of spectra corresponding to the amide I and II regions (1635 and 1531 cm^–1^, respectively), as shown in Figure S2. These results indicate that the presence of PUE may directly influence the hydrogel gelation process, possibly promoting the formation of β-sheet structures. This is supported by the increased intensity of the absorption band at 1531 cm^–1^, which could contribute to enhanced stability of the developed material.^32,33^ Additionally, the appearance of a band at 685 cm^–1^, attributed to amide IV, was observed in the previously SF-G sample, further supporting the conformational changes associated with the gelation process. A broadening of the band around 3300 cm^–1^ was also noted with increasing PUE concentration.

According to Yang et al.,^21^ this phenomenon may be associated with a potential interaction between SF and PUE leading to the formation of a molecular complex, resulting from intermolecular hydrogen bonding during the gelation process. Such interactions are fundamental for the structural organization of hydrogel, directly affecting its physicochemical properties and stability.

The XRD analysis (Figure 1C) revealed that SF exhibited a broad and intense diffraction peak at 22.9°, characteristic of a random coil structure. Additionally, the presence of a low-intensity diffraction peak at 9.5° indicates a small fraction of Silk II crystalline structure, which is consistent with the findings obtained by FTIR.^34^ A diffraction shoulder around 28.2° was also observed, attributed to a minor fraction of Silk I structure.^28^ In contrast, PUE displayed multiple diffraction peaks characteristic of crystalline patterns, located within the 2θ range at 11.1, 14.8, 17.0, 19.9, 22.9, and 26.1°.^31^ When comparing the XRD patterns of SF and PUE with those obtained for the SF+PUE hydrogels, it was observed that the hydrogels exhibited only a broad peak at approximately 22.1°, indicating that the diffraction peaks of SF overlapped with the characteristic peaks of PUE. These results suggest that the presence of PUE in the SF+PUE hydrogel did not significantly alter the crystalline structure of SF, preserving its structural characteristics.^34^ Furthermore, it was possible to observe that the presence of PUE disrupted the crystalline structures associated with the Silk II fraction (9.5°) and the Silk I fraction (28.2°).^35^

### 3.2 Morphological, In Vitro Swelling, and Degradation Properties of Supramolecular Hydrogel

The morphologies of the cryo-fractured cross sections of the lyophilized SF samples, as well as those of the SF hydrogels containing different concentrations of PUE (1–5%), are shown in Figure 2A. SF exhibited a relatively irregular sponge shape morphology characterized by an alveolar structure.^36^ In turn, SEM micrographs of SF+PUE revealed that the cryo-fractured sections displayed a three-dimensional network structure characterized by irregular and interconnected pores. A combination of superficial and deep pores was observed, with variations in wall thickness and a dense arrangement typical of a reticulated network.^35^ Furthermore, it was noted that the gradual increase in PUE concentration promoted the formation of a denser internal network in the SF+PUE hydrogels compared to pure SF. Specifically, samples with lower PUE concentrations (1 and 2%) displayed more well-defined porous networks, whereas those with higher concentrations (3, 4, and 5%) exhibited a more compact structure. Accordingly, the average pore sizes (Figure 2B) for SF+1%PUE, SF+2%PUE, SF+3%PUE, SF+4%PUE, and SF+5%PUE were approximately 91.1 μm (±16.3), 72.2 μm (±7.3), 75.9 μm (±9.0), 56.5 μm (±7.4), and 57.2 μm (9.2), respectively. According to Zhang et al.,^37^ the cross-linking density can directly influence the pore size and morphology of the hydrogel. Furthermore, smaller pore sizes can result in better mechanical strength.^7^ This morphology may enhance nutrient diffusion and provide structural support for cell growth, in addition to contributing to the water absorption capacity - key properties for applications in tissue engineering.^34^ Such structural morphology is particularly advantageous for drug loading and release, thereby expanding the potential biomedical applications of the hydrogel.^38^

**Figure 2:**
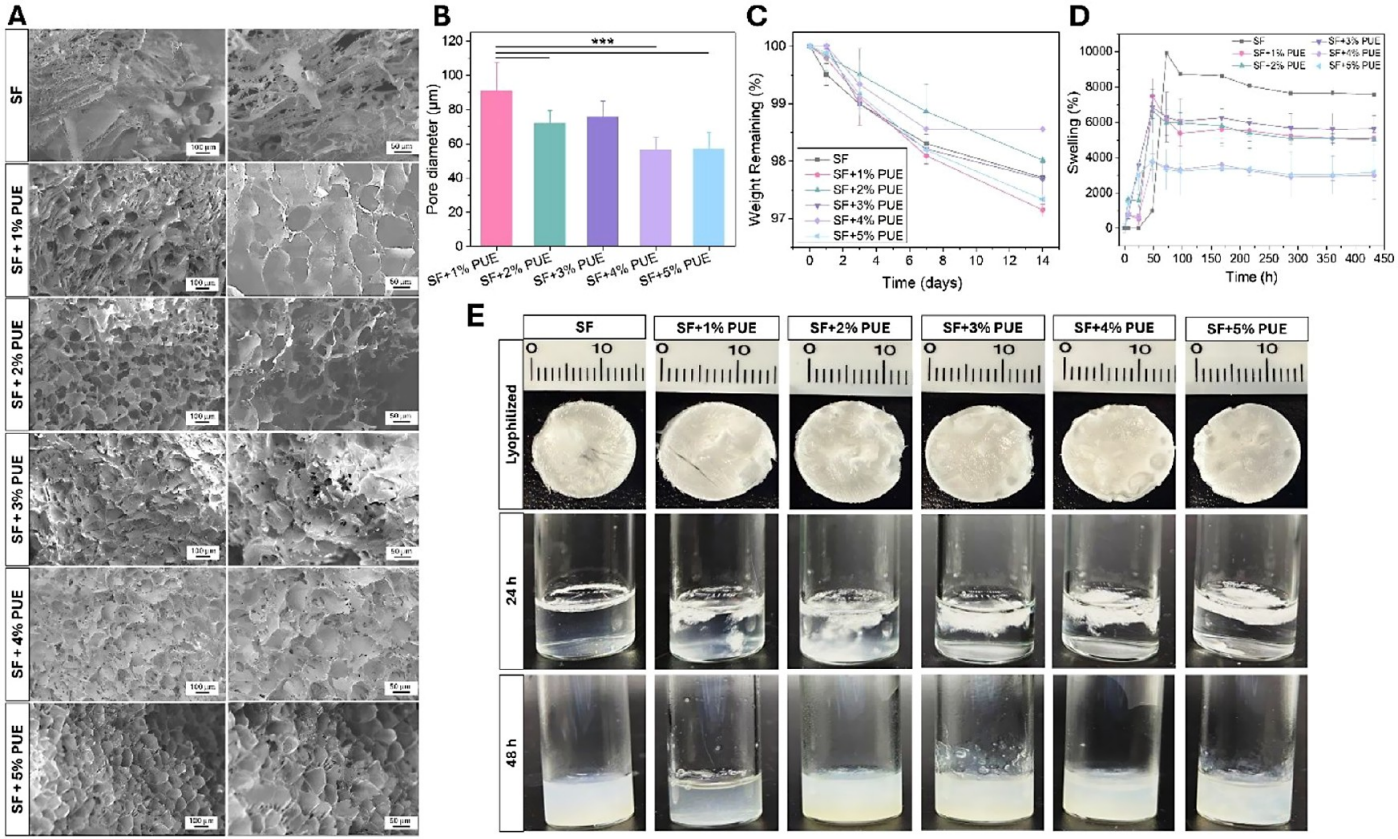
Morphological, swelling, and in vitro degradation properties of SF/PUE hydrogels. (A) Scanning electron microscopy (SEM) images of cryo-fractured SF hydrogels containing different concentrations of puerarin (PUE, 1–5%) at magnifications of 200× and 400× (scale bars: 100 and 50 μm). (B) Quantitative analysis of pore diameter as a function of PUE content. (C) In vitro degradation profiles associated with mass loss of SF/PUE hydrogels over time. (D) Swelling capacity of the hydrogels. (E) Macroscopic evolution of lyophilized SF+PUE hydrogels during rehydration. Statistical significance between groups is ***p < 0.001.

The in vitro degradation study in PBS (Figure 2C) demonstrated that both SF and SF+PUE hydrogels exhibited high stability, retaining approximately 97–99% of their initial mass over a 14-day period. Degradation was measured by quantifying the percentage of mass remaining after incubation of the samples in PBS (pH 7.4, 37 °C), with all values normalized to the initial dry weight. The high mass retention observed indicates limited hydrolytic degradation under simulated physiological conditions. This behavior is consistent with the literature, which reports that silk fibroin-based materials exhibit high resistance to degradation in neutral aqueous environments, attributed to the presence of highly organized β-sheet domains.^39^ Due to its tunable degradation rate and the noncytotoxic nature of its degradation byproducts, silk fibroin offers distinct advantages for biomedical applications, particularly when compared to conventional synthetic and natural polymers.^40^ However, the low degradation observed in PBS also suggests that the material may rely on more complex biological mechanisms for its in vivo degradation, particularly the action of proteolytic enzymes. In this context, enzymatic degradation models are considered more representative for evaluating the behavior of such systems under physiological conditions, as they more accurately mimic the biological environment and provide a better estimation of the material’s biodegradation rate.

All the analyzed formulations exhibited a high apparent swelling capacity throughout the experimental period (Figure 2D). The hydrogel composed solely of SF showed the highest swelling ratio (∼9945%), reaching equilibrium absorption after approximately 72 h. This behavior can be associated with the high density of hydrophilic functional groups within the SF structure, which promote interactions with water molecules.^41^ In addition, the sponge-like porous morphology observed in the SEM micrographs (Figure 2A) may have contributed to this high PBS uptake capacity.

In contrast, the SF+PUE exhibited a concentration-dependent apparent swelling behavior. Formulations containing 1, 2, and 3% PUE (SF+1%PUE, SF+2%PUE, and SF+3%PUE) reached a maximum apparent swelling value of approximately 6814% after 48 h. However, samples with higher PUE content (SF+4%PUE and SF+5%PUE) showed a reduced swelling capacity compared to the other groups, reaching a plateau of approximately 3737% at 48 h. This reduction is likely associated with a more compact microstructure, as evidenced by the smaller pore sizes in SEM analysis, as well as an increased density of physical interactions within the hydrogel network. As reported by Xu et al.,^42^ more densely organized networks with smaller pore sizes hinder water diffusion into the matrix, resulting in a reduced swelling capacity. Similarly, Amirian et al.^43^ observed comparable behavior in photo-cross-linkable composite hydrogels based on methacrylated silk fibroin and methacrylated hyaluronic acid, which exhibited high swelling ratios. According to the authors, the swelling capacity of a material is directly related to its internal structure and is typically higher in systems with lower cross-linking density and greater porosity, which facilitate water absorption.

The swelling behavior of hydrogels is a critical parameter, particularly for biomedical applications, as it can directly influence drug delivery, as well as the transport of nutrients and growth factors. Consequently, it affects cellular responses such as proliferation and differentiation and plays a key role in controlling the release kinetics of active agents, which is largely governed by matrix swelling.^44^ According to Ou et al.,^45^ in chronic wounds, the excessive accumulation of exudate promotes bacterial growth, which can lead to infections and delay the healing process. Therefore, hydrogels with high swelling capacity have the potential to absorb excess wound exudate, helping to maintain a controlled moist environment and consequently promoting tissue regeneration. Furthermore, as highlighted by Feng and Wang,^46^ hydrogels exhibiting high swelling capacity are widely explored in tissue engineering and drug delivery applications

It is important to highlight that the swelling values reported herein may reflect not only water uptake but also structural changes occurring during rehydration, including expansion, partial disintegration, and network rearrangement of the lyophilized matrix. The macroscopic evolution of hydrogels during rehydration is presented in Figure 2E. After lyophilization, all samples exhibited a well-defined porous structure. The lyophilized hydrogels were immersed in 2 mL of PBS, and the macroscopic changes were monitored over time. Upon immersion, the hydrogels rapidly absorbed the medium, resulting in significant volumetric expansion within the first 24 h. However, this process was accompanied by progressive loss of structural integrity, particularly for formulations with lower PUE content. After 48 h, all samples formed a continuous gel-like phase that adapted to the shape of the container, indicating extensive structural rearrangement of the network. These observations suggest that the swelling process involves not only water uptake but also partial dissolution and reorganization of the polymeric matrix, which is consistent with the dynamic nature of physically cross-linked supramolecular hydrogels. Notably, these macroscopic findings agree with the swelling results (Figure 2D), confirming that the high swelling values are associated with both water absorption and structural transformation of the lyophilized matrix.

The results suggest that the SF+PUE system exhibits behavior characteristic of a dynamic hydrogel, as the formed network displays a soft viscoelastic nature, high rehydration capacity, and evident structural reorganization in aqueous environments. This behavior is consistent with physically cross-linked networks, in which reversible interactions enable structural adaptation and rearrangement over time. These dynamic processes facilitate energy dissipation within the hydrogel matrix, thereby imparting injectability and self-healing properties that are crucial for clinical applications.^47^ In addition, the swelling results corroborate the FTIR findings (Figure 1B), which revealed the formation of hydrogen bonding interactions between SF and PUE, further supporting the role of these interactions in governing the organization and dynamic behavior of the supramolecular network.

### 3.3 Thermal Analysis

The thermal stability of PUE, lyophilized SF, and hydrogels formulated with SF and increasing concentrations of PUE (1–5%), was evaluated by thermogravimetric analysis (TGA) and its derivative curve (DTG) (Figure 3A), as well as by differential scanning calorimetry (DSC) (Figure 3B).

**Figure 3:**
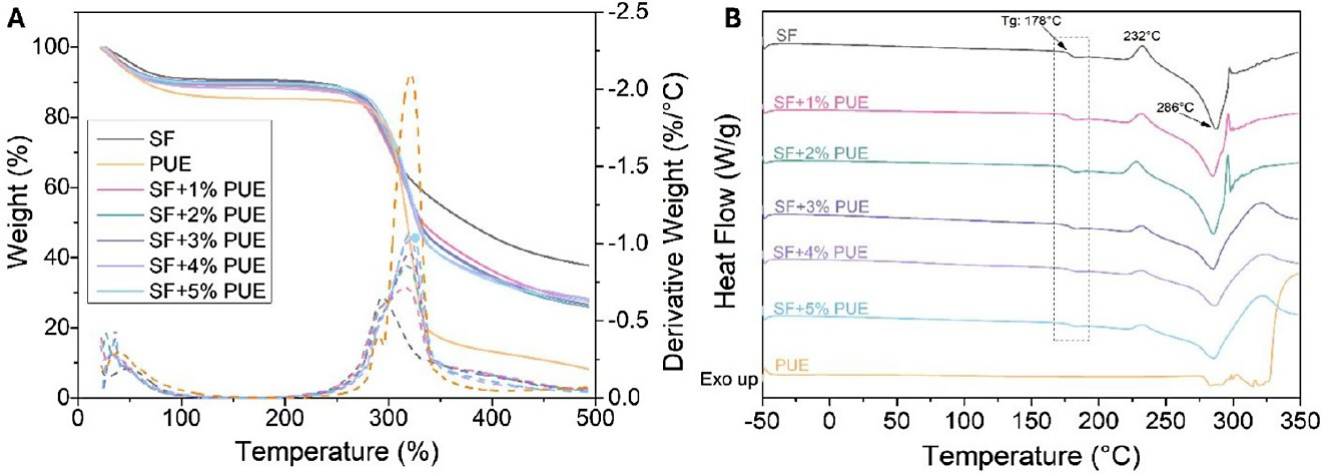
Thermal analysis. (A) TGA/DTG and (B) DSC curves of lyophilized silk fibroin (SF), puerarin (PUE), and lyophilized SF hydrogels with different concentrations of PUE (1–5%).

The TGA (Figure 3A) revealed distinct degradation profiles for the materials studied, as summarized in Table S1. SF exhibited two main mass loss events. The first event, corresponding to an 8% mass loss, occurred in the temperature range of 24 and 70 °C, with a maximum mass loss temperature (*T*_max_) at 47 °C, attributed to moisture loss. The second and more pronounced stage was observed between 275 and 430 °C, presenting a *T*_max_ at 294 °C and a mass loss of about 53%, associated with the breakdown of the polymer chain length and the cleavage of peptide bonds in SF.^48^ In contrast, PUE powder exhibited three distinct thermal degradation events. The first stage resulted in a mass loss of approximately 13% within the temperature interval of 30 and 77 °C, with a *T*_max_ at 38 °C, and is attributed to the evaporation of free and adsorbed water molecules.^49^ The second and third events occurred sequentially, resulting in mass losses of 10 and 67%, respectively. The second event took place between 281 and 294 °C, with a *T*_max_ at 290 °C, while the third event occurred between 300 and 334 °C, with a *T*_max_ at 322 °C. Both events were attributed to the thermal degradation of the molecular structures of PUE.^50^

The SF+PUE hydrogel formulations exhibited two distinct mass loss events. The first event, accounting for approximately 10% mass loss, occurred at temperatures below 100 °C, with a *T*_max_ around 34 °C, and was attributed to the release of water retained within the material. The second, more pronounced event took place between 275 and 335 °C, featuring two consecutive stages of mass loss, as evidenced by the DTG curve (Figure 3A). The hydrogels exhibited *T*_max_ values at approximately 293 and 320 °C. Accordingly, the SF+1%PUE, SF+2%PUE, SF+3%PUE, SF+4%PUE, and SF+5%PUE hydrogels showed mass losses of 61, 63, 63, 59, and 59%, respectively. This thermal event was associated with the combined thermal degradation of SF and PUE.

The DSC analysis (Figure 3B) shows the second heating scan. Accordingly, the DSC curve of SF exhibited a glass transition temperature (*T*_g_) at 178 °C, corroborating the findings reported by Pham et al.^51^ Additionally, an exothermic peak at approximately 232 °C was detected, which is associated with the formation of β-sheets crystalline structures (Silk II). A subsequent broad endothermic event around 286 °C was associated with the thermal decomposition of SF.^52^ In contrast, the DSC curve of PUE showed only a single endothermic event within the temperature range of 276 to 326 °C, corresponding to its melting point.^53^ The DSC curves of SF-based hydrogels incorporated with different concentrations of PUE (1–5%) displayed thermal profiles similar to that of pure SF, with no variation in *T*_g_. In turn, the same characteristic endothermic and exothermic events of SF were observed, accompanied by a decrease in the exothermic peak associated with the crystallization of SF (232 °C) as the PUE content in the hydrogel gradually increased (Δ*H*_SF_: 13.5 J/g; Δ*H*_SF+1%PUE_: 8.5 J/g; Δ*H*_SF+2%PUE_: 9.6 J/g; Δ*H*_SF+3%PUE_: 4.8 J/g; Δ*H*_SF+4%PUE_: 3.9 J/g; Δ*H*_SF+5%PUE_: 3.9 J/g). Furthermore, thermal events related to the melting of PUE were not detected in the SF+PUE system, supporting the findings of the XRD analysis (Figure 1C), which indicate that the incorporation of PUE into SF leads to a loss of crystalline organization.

### 3.4 Rheological Performance and Applicability of Hydrogels

The vial inversion test (Figure 4A) demonstrates the formation of a turbid gel following the cooling process, as well as its structural stability. It was observed that increasing the concentration of PUE in SF promotes the formation of more stable hydrogels, as evidenced by the formulations containing 4 and 5% PUE (SF+4%PUE and SF+5%PUE).

**Figure 4:**
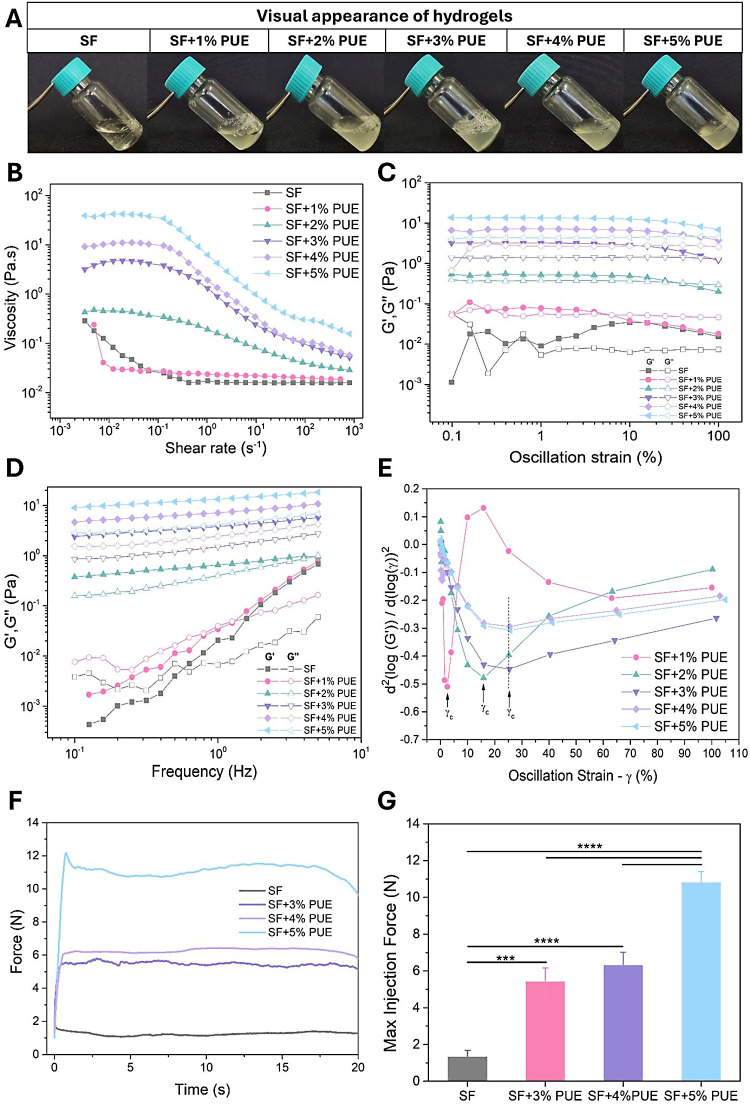
Analysis of silk fibroin (SF) solution and SF hydrogels containing different concentrations of PUE (1–5%). (A) Apparent viscosity of the hydrogels after the heating and cooling process. (B) Rheological analysis of viscosity as a function of shear rate. (C) Oscillatory shear stress sweep. (D) Rheological evaluation of viscoelastic properties based on storage (*G*′) and loss (*G*″) moduli. (E) Second derivative curves of the *G*′ as a function of oscillation strain. (F) Injectability test. (G) Quantification of maximum injection force. Statistical significance is indicated by the symbols *p < 0.05, **p < 0.01, ***p < 0.001, and ****p < 0.0001.

Rheological analyses were conducted to evaluate the properties of the hydrogels regarding their structure and mechanical behavior. Viscosity curves as a function of shear rate (Figure 4B) demonstrate that the viscosity of the hydrogels increases proportionally with the concentration of PUE present in the SF+PUE system, corroborating the vial inversion test results (Figure 4A). The increase in viscosity with higher PUE content in SF suggests possible physical cross-linking between both components, as well as strengthening of the polymer network, characteristic of shear thinning. According to Daneshvar et al.^54^ this behavior can be advantageous for practical applications such as injectable systems, as it allows the material to flow easily under shear stress while maintaining its structural viscosity once the stress is removed. While the SF solution exhibits low viscosity, the samples composed of SF+3% PUE, SF+4% PUE, and SF+5% PUE showed a significant increase in viscosity at low shear rates. This behavior decreases with an increasing shear rate, revealing inherent pseudoplastic behavior. The gelation kinetics evaluated by time sweep analysis (Figure S3) demonstrated that the sample composed solely of SF exhibited a loss modulus (*G*″) higher than the storage modulus (*G*′), indicating a typical sol-like behavior. In contrast, the progressive incorporation of PUE into the formulation resulted in the predominance of *G*′ over *G*″ in the SF+PUE hydrogels. This behavior indicates that the SF+PUE hydrogels exhibited a predominantly elastic (gel-like) behavior from the beginning of the experiment, with no observable sol–gel transition throughout the entire time sweep.^55,56^

The response of the hydrogels to oscillatory shear stress was evaluated through the *G*′ and *G*″ moduli as a function of angular frequency, allowing the assessment of the mechanical strength of the polymeric network in the SF+PUE hydrogels. To minimize potential wall slip effects during measurements, the linear viscoelastic region (LVR) of the samples was initially determined (Figure 4C). Based on these results, a strain of 1% was selected for the subsequent analyses. As shown in Figure 4D, both the SF solution and SF+1%PUE displayed sol–gel behavior. With increasing PUE concentration (≥3%), *G*′ surpassed *G*″ across all frequencies, indicating the formation of a stable, solid-like viscoelastic network characteristic of SF-PUE hydrogels.^57^ This phenomenon is associated with predominantly elastic behavior.^48^ These results suggest an enhancement of the mechanical properties of SF hydrogels upon PUE addition.^58^ According to Pang et al.,^13^ a significant increase in both *G*′ and *G*″ moduli with increasing PUE concentration in the system is expected, supporting the findings of this study. This behavior can also be attributed to the physical interactions between SF and PUE, resulting in increased mechanical strength of the hydrogels.^59^ Furthermore, the increase of *G*′ relative to *G*″ indicates an increase in β-sheet content within the hydrogel structure, as evidenced by FTIR analysis (Figure 1B).^48^ Importantly, these structural changes are consistent with the proposed mechanism in which PUE promotes β-sheet formation through noncovalent interactions. This interpretation is further supported by the observed reduction in pore size (Figure 2A,B) and the increase in *G*′ (Figure 4D), suggesting the formation of a more densely cross-linked network in the presence of PUE. For biomedical applications, injectable hydrogels should exhibit fluid-like behavior during injection and solidify after ejection (*G*′ > *G*″), consistent with the results presented herein.^60^ Similarly, Ni et al.^61^ developed SF hydrogels combined with the polysaccharide alginate. According to the authors, the hydrogel not only serves as a platform for exosome release but also provides mechanical support to the infarcted myocardium.

The second derivative of *G*′ as a function of oscillatory stress for the hydrogels (SF+PUE) exhibits a valley corresponding to the critical strain point (γ_c_), which marks the limit of the linear viscoelastic region (LVR) and reflects the structural strength of the gel (Figure 4E). It was observed that the γ_c_ values varied according to the incorporated PUE concentration, being 2.5% for SF+1%PUE, 15.9% for SF+2%PUE, 25.4% for SF+3%PUE, 25.5% for SF+4%PUE, and 25.7% for SF+5%PUE. In this case, higher PUE concentrations resulted in higher γ_c_ values. This behavior can be attributed to stronger interactions between SF and PUE chains in samples with higher PUE contents (2–5%), leading to a mechanically more robust structure compared to the sample with lower PUE concentration (SF+1%PUE).^7^ However, for the samples containing 3, 4, and 5% PUE, the γ_c_ values did not differ significantly (approximately 25%), indicating the presence of a maximum concentration effect of PUE, above which no further improvement in the structural strength of the hydrogel is observed. This behavior may be related to the frequency sweep test, which subjects the samples to high-frequency values that can override the interactions between SF and PUE, even when these interactions are stronger, thereby leading to the disruption of the hydrogel structure.

In turn, the mechanical strength (*G*′) values of the hydrogel as a function of PUE concentration (Figure S4A) show that at low PUE contents (SF+1%PUE and SF+2%PUE), the hydrogel exhibits low strength, with values of 2.1 and 1.0 Pa, respectively. However, the SF+3%PUE, SF+4%PUE, and SF+5%PUE formulations displayed values of 6.3, 13.9, and 21.5 Pa, respectively, indicating an increase in mechanical strength with the rise in PUE concentration within the system. This enhanced stiffness may be associated with stronger interactions between the SF and PUE chains, leading to reduced chain mobility. Finally, the *G*′ values at maximum and minimum frequencies as a function of PUE concentration were evaluated (Figure S4B). When comparing the same sample, it is possible to observe that at the maximum frequency, the G’ value is consistently higher than that observed at the minimum frequency. This occurs because, at lower frequencies, the deformations within the hydrogel structure take place more slowly, allowing sufficient time for the polymeric network to relax the applied stress. Consequently, the mechanical resistance of the hydrogel is lower in the low-frequency region. Furthermore, it can be observed that the SF+5%PUE sample exhibited the highest mechanical strength at the lowest frequency region, indicating stronger interactions between the SF and PUE chains within the hydrogel. Such interactions hinder the stress relaxation process in the polymeric network, corroborating the analysis presented in Figure S4A.^62^ These results are consistent with the morphological analysis (Figure 2A,B), which shows that samples with higher PUE concentrations (4 and 5%) displayed smaller pore sizes, a feature that may contribute to the enhancement of mechanical resistance against external forces.^7^

Similar rheological results were reported by Chen et al.,^56^ in which self-assembled hydrogels based on PUE and chitosan exhibited viscoelastic properties suitable for applications in skin wound healing, particularly for chronic infected wounds. Likewise, the rheological results obtained for the SF+PUE hydrogels indicate the formation of a soft viscoelastic network. This behavior is characteristic of physically cross-linked supramolecular hydrogels and is particularly desirable for biomedical applications that require high mechanical compliance. In this context, soft supramolecular hydrogels have been widely explored for applications such as controlled drug delivery, tissue engineering, and wound healing.^63^

Additionally, the injectability of these hydrogels was assessed using a 27G needle (Figure 4F). The SF solution without the addition of PUE did not exhibit continuous extrudability, showing irregular dripping when pressed through the syringe, indicating limited injectability under the evaluated experimental conditions. With the incorporation of PUE, a change in flow behavior was observed, enabling progressive extrusion of the material through the needle with measurable and reproducible forces. Specifically, as measured in Figure 4G, the injection force increased from 1.31 N (±0.37 N) for SF to 5.49 N (±1.02 N) for SF+3% PUE, remaining similar for SF+4% PUE (6.05 N ± 0.78 N), and reaching 10.79 N (±0.61 N) for SF+5% PUE. The load–displacement curve exhibited a peak force followed by a stable plateau, characteristic of non-Newtonian materials undergoing flow adjustment under constant shear.^64^ These results indicate that the addition of PUE enables the system to be extruded through a fine needle (27 G), in contrast to SF alone. Although the injection force values increase with PUE content - likely due to higher viscosity and stronger supramolecular interactions - the magnitude of the observed forces is consistent with injectable hydrogels reported in the literature, which typically require forces below ∼15–20 N for manual extrusion through small-gauge needles, depending on testing conditions (e.g., extrusion speed, needle length, and system geometry).^65^ Furthermore, rheological analysis demonstrates that SF+PUE hydrogels exhibit shear-thinning behavior, as evidenced by the decrease in viscosity with increasing shear rate. This behavior facilitates flow under applied stress during injection, thereby compensating for the relatively high zero-shear viscosity and contributing to the overall injectability of the system. Moreover, as shown in Figure 4B, the hydrogels display enhanced flowability at higher shear rates, which further supports their suitability for injectable biomedical applications.^41^ In turn, the predominance of *G*′ over *G*″ indicates a solid-like network capable of recovering its structural integrity after extrusion. Accordingly, the observed increase in injection force reflects improved network formation and mechanical stability, while the shear-thinning behavior enables efficient flow under injection conditions. The balance between these factors results in superior functional injectability compared to SF alone. These results suggest that the evaluated formulations exhibit good injectability for clinical application.^66^

### 3.5 In Vitro Biological Characterization

#### 3.5.1 Cytocompatibility

In vitro cytotoxicity assays were conducted by evaluating metabolic activity and cell viability (live/dead), using human dermal fibroblasts (HDF) at 1, 3, and 6 days. These experiments were designed to investigate biocompatibility, as well as the ability of the materials to support cell viability and proliferation following indirect exposure to SF and SF+PUE hydrogels containing different PUE concentrations (1–5%). For this purpose, an indirect culture system based on Transwell inserts was employed, in which HDF cells were seeded in the lower compartment, while the hydrogel samples were placed in the upper insert. This configuration allows the diffusion of soluble factors released from the materials without direct physical contact with the cells, enabling the assessment of potential cytotoxic or bioactive effects mediated by released compounds. A schematic representation of this experimental setup is presented in Figure 5A.

**Figure 5:**
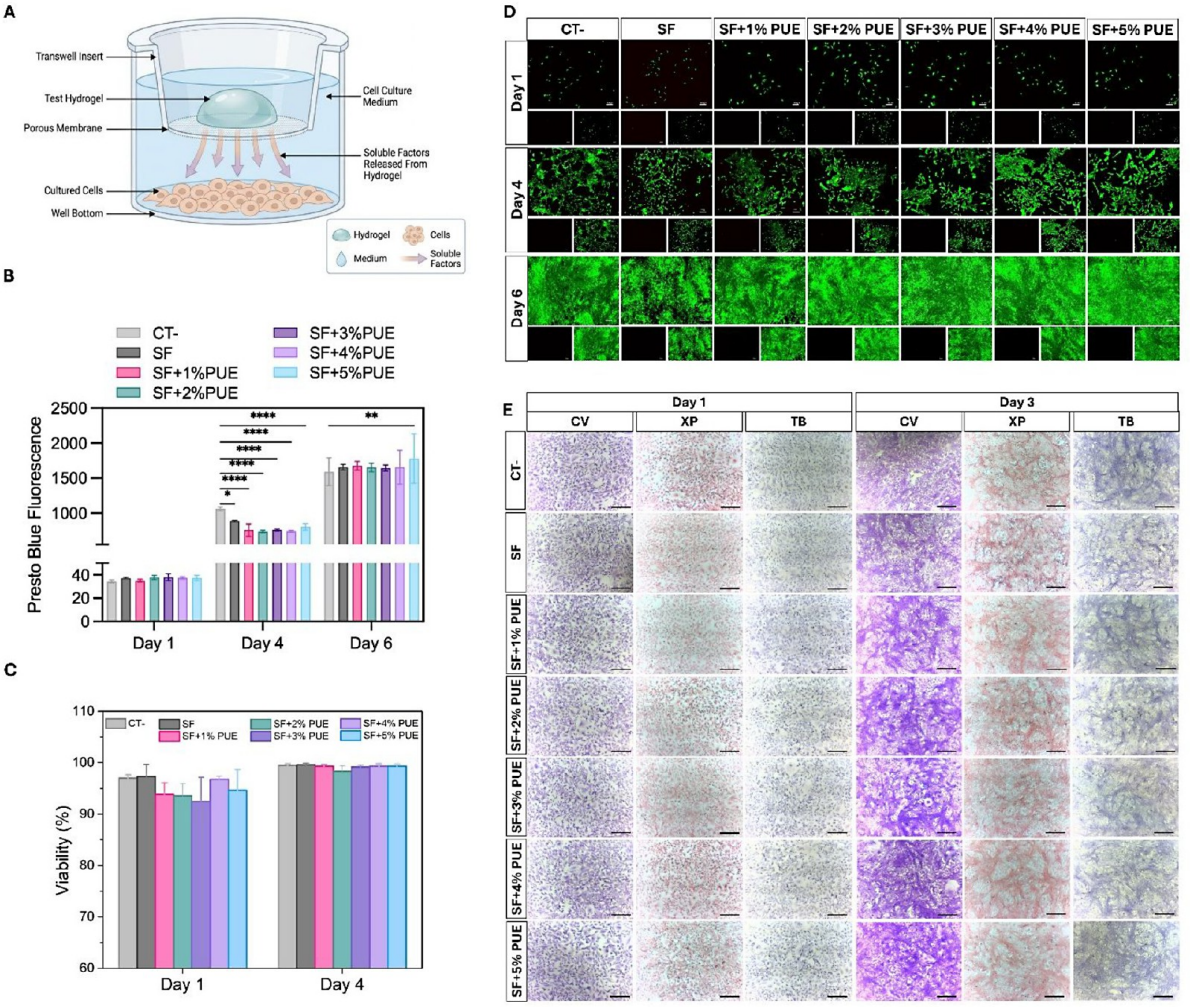
In vitro cytotoxicity evaluation of cells in contact with silk fibroin (SF) solution and SF hydrogels containing different concentrations of puerarin (PUE) (1–5%). (A) Schematic illustration of the indirect culture system using Transwell inserts. (B) PrestoBlue assay, showing cell viability and metabolic activity of HDF cells. (C) Quantification of live and dead cells. (D) Live/Dead viability assay of HDF cells in contact with the hydrogels (scale bar: 100 μm). (E) Cytological staining of Vero cells after incubation with the hydrogels using cresyl violet (CV), toluidine blue (TB), and xylidine ponceau (XC). The negative control (CT-) corresponds to cells cultured in the absence of hydrogels (Scale bar: 50 μm). Statistical significance between groups is indicated by *p < 0.05, **p < 0.01, and ****p < 0.0001.

The metabolic activity results (Figure 5B) showed a progressive increase in cell proliferation over the experimental period. By day 6, statistically significant differences (*p* < 0.01) were observed between the sample containing the highest PUE concentration (SF+5%PUE) and the negative control (CT-), corresponding to cells cultured in the absence of hydrogels. Overall, the findings indicate that the formulations are noncytotoxic and capable of supporting cell proliferation. Furthermore, the incorporation of higher PUE concentrations appears to positively modulate cellular activity, suggesting that the SF+PUE system, particularly at 5% PUE, has potential to enhance cell proliferation without compromising biocompatibility.

For cell viability assessment, HDF cells were stained with calcein-AM (green fluorescence) to identify viable cells and ethidium homodimer-1 (red fluorescence) to detect nonviable cells. Quantitative analysis (Figure 5C), based on fluorescence microscopy images (Figure 5D), demonstrated high cell viability at all time points, with a predominance of viable cells, comparable to the negative control, and with almost no dead cells observed. Additionally, cell adhesion and proliferation were observed throughout the culture period, with cell viability exceeding 95% as early as day 1 (Figure 5C). No significant differences in the number of viable cells were detected among the experimental groups, indicating the absence of cytotoxic effects in the tested formulations and corroborating the results obtained from the metabolic activity assay (Figure 5B).

The in vitro cytotoxicity was evaluated in Vero cells exposed to extracts of SF and SF+PUE hydrogels (Figure 5E) using specific histological stains to assess cellular morphology and integrity. The nonspecific dye Crystal Violet (CV) was used to assess cell morphology, while the acidic dye Xylidine Ponceau (XP) indicated the presence of cationic radicals associated with proteins. In turn, Toluidine Blue (TB), a basic dye that forms electrostatic bonds with acidic radicals in tissues, revealed the presence of deoxyribonucleic acid (DNA), ribonucleic acid (RNA), and glycosaminoglycans.^24,67,68^

Direct cytotoxicity analysis after 24 h revealed that the CT- group exhibited cells with typical epithelial morphology, forming a confluent monolayer. The other samples (SF, SF+1%PUE, SF+2%PUE, SF+3%PUE, SF+4%PUE, and SF+5%PUE) showed a similar morphological pattern to the CT- group, characterized by the presence of elongated cells. This behavior indicates cell adhesion and compatibility with the tested hydrogels. However, after 72 h, an apparent increase in cell density was observed, accompanied by disorganized growth and the formation of overlapping cell layers. These findings are consistent with the exponential increase in cell viability detected in the Presto Blue assay performed during the same period (Figure 5B). Notably, no significant cytoplasmic alterations or morphological features indicative of cell death were observed under any of the tested conditions.

SF is a natural biopolymer widely recognized for its biocompatibility and lack of inflammatory responses, in addition to showing no cytotoxic effects across various cell lines. According to findings by Zhang et al.^12^ SF-based hydrogels possess great potential for a wide range of applications in tissue engineering, including bone, cartilage, skin, nerves, blood vessels, ligaments, tendons, liver, cornea, tympanic membrane, teeth, bladder, among other organs. Additionally, Ni et al. (2024)^61^ reported that hydrogels composed of SF and alginate significantly increased cell viability in H9c2 cell lines, highlighting their potential use in cardiac therapies, such as myocardial infarction.

PUE, in turn, exhibits several pharmacological activities, including antioxidants, anti-inflammatory, and collagen-inducing effects.^69^ Studies conducted by Dou et al. (2023)^50^ showed that treatment with low-molecular-weight PUE promoted increased viability of HepG2 cells by protecting against palmitic acid-induced lipotoxicity. Similarly, Gao et al.^70^ reported that isoflavonoids such as PUE can protect human umbilical vein endothelial cells from reactive oxygen species (ROS)-induced apoptosis, a critical factor in vascular cell dysfunction. According to the authors, PUE is associated with antioxidant, cardioprotective, neuroprotective, anticancer, and anti-inflammatory effects. Furthermore, Fan et al.^71^ emphasized the cardioprotective effects of PUE, including increased cell viability and suppression of inflammatory processes in acute myocardial infarction models.

According to the criteria established by ISO 10993–5, the obtained results indicate that the developed hydrogels are noncytotoxic and capable of promoting and stimulating cell proliferation. Moreover, the findings suggest an effect associated with the presence of PUE, reinforcing the potential of SF+PUE formulations for biomedical applications, particularly in the context of tissue regeneration.

#### 3.5.2 Scratch Assay

Figure 6A illustrates the schematic of the wound healing assay performed in Vero cells. After the formation of a confluent cell monolayer, a linear scratch was created on the culture surface using a 200 μL pipet tip, simulating an in vitro wound. The cells were then incubated with SF-based hydrogels containing different concentrations of PUE (1–5%) for a period of 72 h, allowing the evaluation of cell migration and wound closure. This assay is widely used as an in vitro method to investigate wound healing potential.^72^ Wound healing efficiency was quantified as the percentage of closure over time at 24, 48, and 72 h (Figure 6B), and representative optical microscopy images illustrating the progressive closure of the wound are presented in Figure 6C.

**Figure 6:**
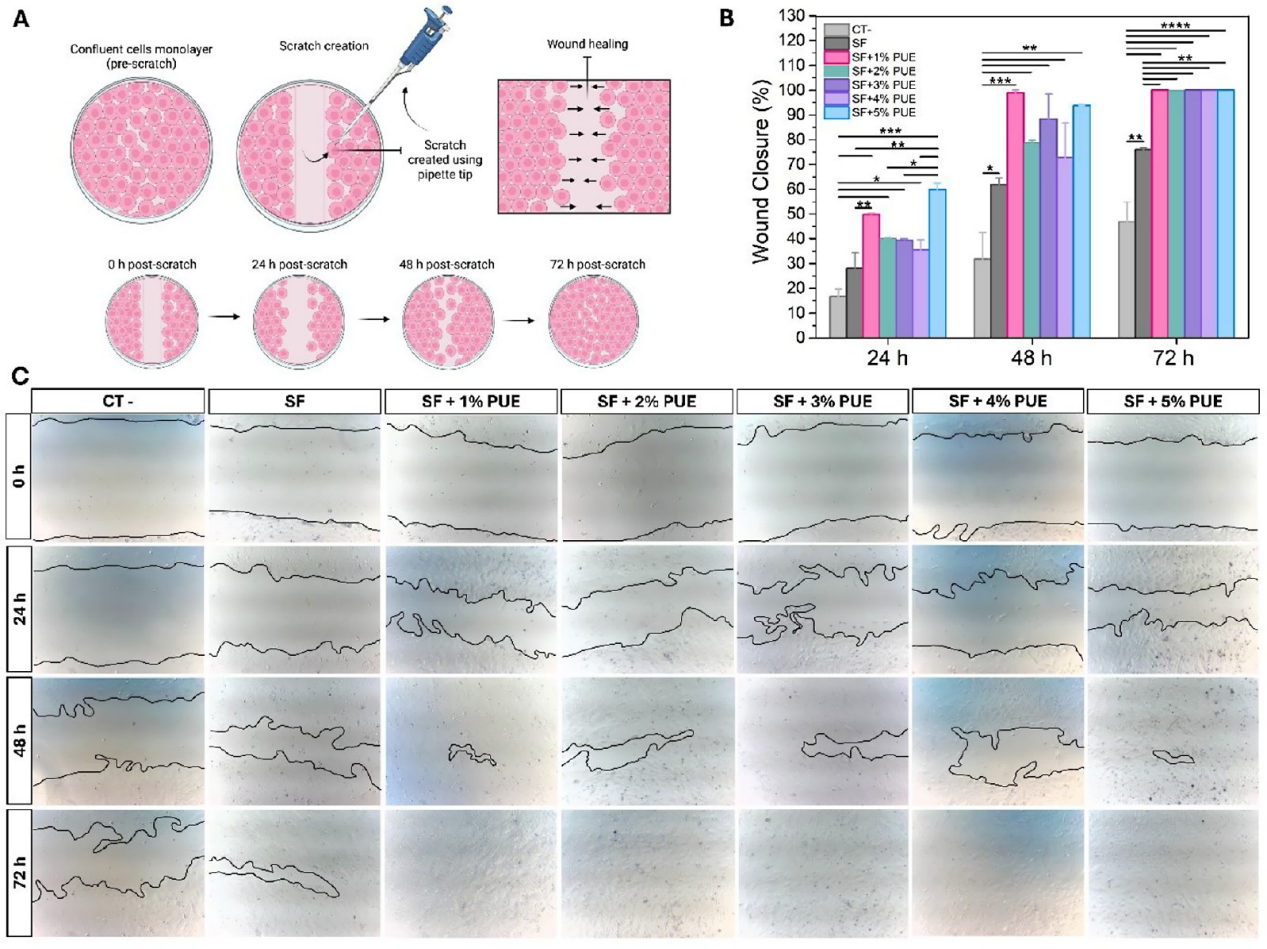
Scratch assay. (A) Schematic illustration of the scratch assay performed using a 200 μL pipet tip, showing the initial wound formation and its closure after 72 h; (B) graph showing the percentage of wound closure over time; and (C) optical microscopy micrographs illustrating cell migration and in vitro wound closure in the presence of silk fibroin (SF) extract and SF-based hydrogels containing different concentrations of PUE (1–5%). The negative control (CT-) corresponds to cells cultured in the absence of hydrogels. Analyses were performed at 0, 24, 48, and 72 h. Statistical significance between groups is indicated by *p < 0.05, **p < 0.01, ***p < 0.001, and ****p < 0.0001.

At all analyzed time points (24, 48, and 72 h), cells in contact with the extracts of SF+PUE hydrogels exhibited a progressive and significant increase in wound closure percentage when compared to the negative control (CT-) and to SF alone, indicating a time-dependent effect (Figure 6B,C). After 24 h, the SF+5% PUE formulation promoted approximately 60% wound closure, a value higher than that observed for the other samples. At 48 h, the SF+1% PUE sample demonstrated a remarkable wound closure capacity, reaching approximately 98%, a result comparable to that of SF+5% PUE (94%). During this period, all SF+PUE formulations showed statistically significant differences only when compared to the CT- group. However, after 72 h, complete cell confluence and full wound closure (100%) were observed in all SF+PUE samples, regardless of PUE concentration. These results revealed notable cell adhesion, viability, and proliferation in the presence of SF+PUE hydrogels. At this stage, the hydrogels showed statistically significant differences (*p* < 0.01) compared to SF, and highly significant differences (*p* < 0.001) compared to the CT- group, which reached only about 46% wound closure. These findings are consistent with the cell viability assay results and reinforce the potential of SF+PUE hydrogels as promising biomaterials for biomedical applications.

## 4 Conclusions

The presence of PUE into SF hydrogels promoted intermolecular interactions that enhanced gelation without altering the crystalline structure of SF. Morphological changes resulted in reduced porosity and swelling, while rheological evaluations demonstrated increased viscosity and viscoelastic moduli, indicating the formation of a reinforced, physically cross-linked network. These features improved injectability and gelation behavior, favorable for minimally invasive applications. Thermal analyses confirmed the structural stability of the hybrid system. The in vitro degradation test demonstrated that the hydrogel remained stable over a 14-day evaluation period. In vitro assays demonstrated excellent cytocompatibility, adhesion, and complete wound closure, supporting the biological safety and efficacy of the material. Altogether, the SF+PUE injectable hydrogels showed suitable physicochemical, mechanical, and biological characteristics, underscoring their potential for applications in tissue engineering and regenerative medicine. Although SF+PUE hydrogels have demonstrated promising physicochemical, mechanical, and biological properties in vitro, further studies are warranted, including DNA quantification assays and in vivo models, to comprehensively evaluate their biocompatibility, degradation behavior, and therapeutic efficacy under more complex physiological conditions.

## Supplementary Material



## Data Availability

Data will be made available on request.

## Notes

Macroscopic images of silk fibroin and puerarin supramolecular hydrogels, deconvoluted FTIR spectra, table containing thermal events obtained from thermogravimetric analysis, and complementary rheological data (PDF)
