# Supplementary material for: Injectable Silk Fibroin–Puerarin Hydrogels with Tunable Supramolecular Organization as a Potential Platform for Tissue Engineering
Source: ACS Omega. 2026 May 13;11(20):30100–15. doi: 10.1021/acsomega.6c02412 (PMC13216943; doi:10.1021/acsomega.6c02412)
Supplement: Supplementary file 1 [file ao6c02412_si_001.pdf]

Supporting Information

Injectable Silk Fibroin-Puerarin Hydrogels with Tunable Supramolecular Organization as a Potential Platform for Tissue Engineering

Bruna V. Quevedo[a,b,c*], Bianca Sabino Leocádio Antunes[a,b], Saeed Safari[c], David Hubbard[c], Daniel Komatsu[b], Menekse Ermis [c], Eliana Aparecida de Rezende Duek[a,b]

[a]Postgraduate Program in Materials Science (PPGCM), Federal University of São Carlos (UFSCar), Sorocaba, SP, 18052-780, Brazil.

[b]Laboratory of Biomaterials, Faculty of Medical Sciences and Health (FCMS), Pontifical Catholic University of São Paulo (PUC-SP), Sorocaba, SP, 18030-070, Brazil.

[c]Terasaki Institute for Biomedical Innovation (TIBI), Los Angeles, CA, 91367, USA.

*E-mail: brunaquevedo@estudante.ufscar.br

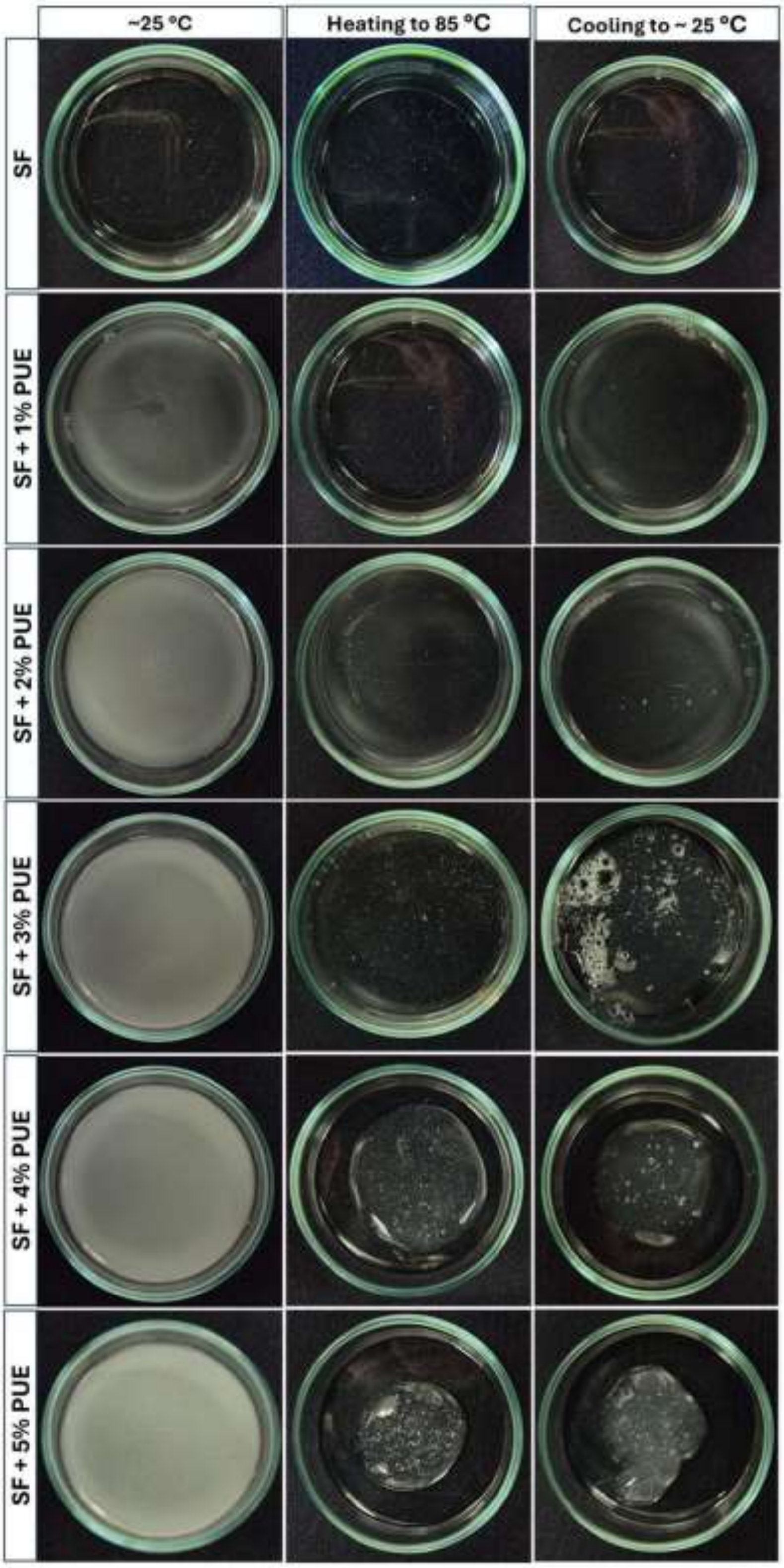

Fig. S1. Hydrogel formation. Images of hydrogel formation by self-assembly after heating and cooling of the silk fibroin (SF) solution containing different concentrations of puerarin (PUE) (1- 5%).

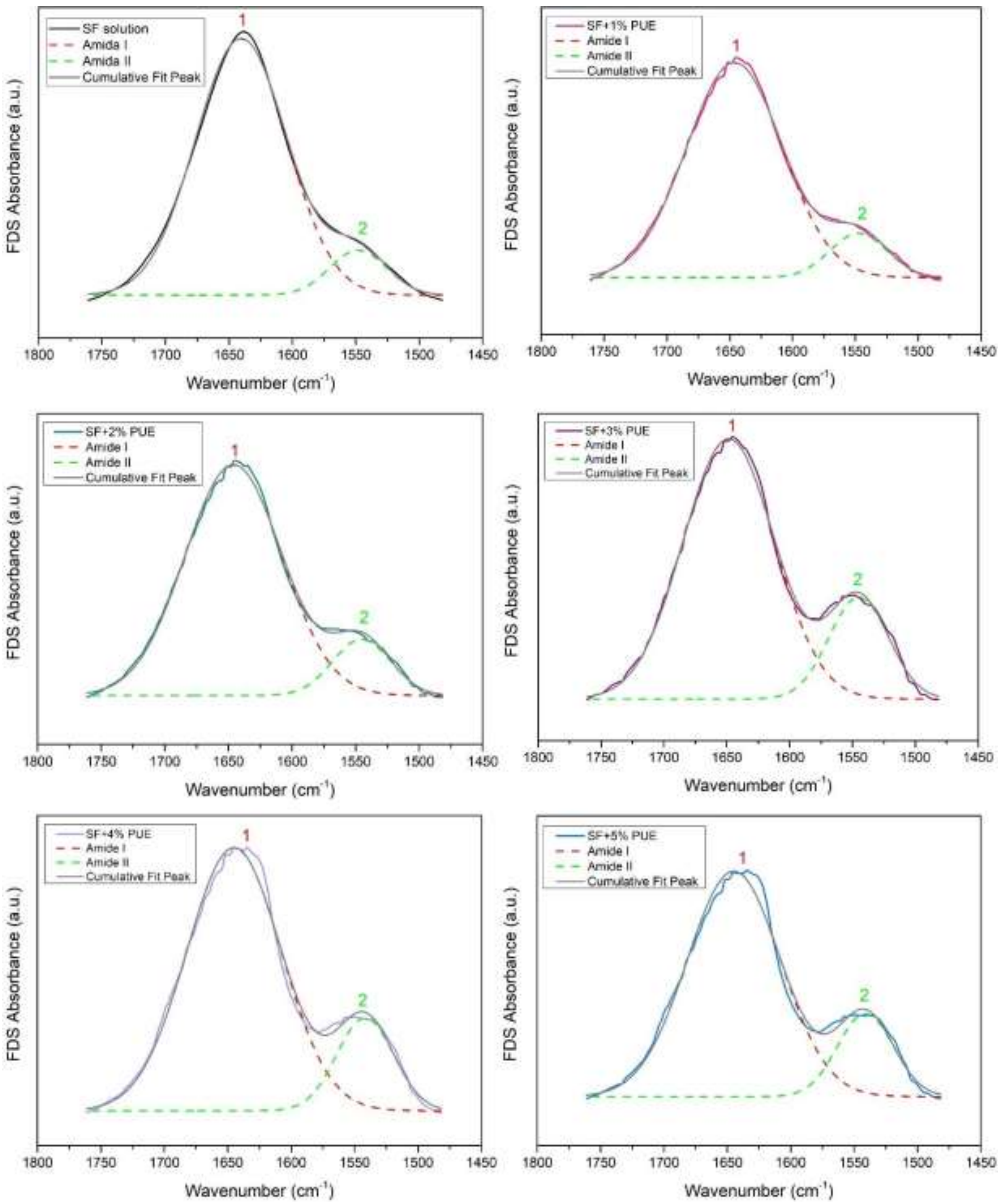

Fig. S2. FTIR deconvolution. Deconvolution of FTIR spectra in the regions corresponding to the peaks at 1635 cm^{-1} (amide I) and 1531 cm^{-1} (amide II) of silk fibroin (SF) and SF-based hydrogels containing different concentrations of PUE (1-5%).

Table S1. Thermal properties. TGA and DTG analyses lyophilized samples of silk fibroin (SF), Puerarin (PUE), and lyophilized SF hydrogels containing different concentrations of PUE (1-5%).

Sample	Events	T_{onset} (°C)	T_{endset} (°C)	T_{max} (°C)	Weight loss (%)
**SF**	**1**	24	70	47	8
	**2**	275	430	294	53
**PUE**	**1**	30	77	38	13
	**2**	281	294	290	10
	**3**	300	334	322	67
**SF+1% PUE**	**1**	23	59	34	10
	**2**	274	335	293/317	61
**SF+2% PUE**	**1**	28	64	35	10
	**2**	273	336	294/318	63
**SF+3% PUE**	**1**	31	73	35	10
	**2**	276	338	293/320	63
**SF+4% PUE**	**1**	22	67	34	10
	**2**	278	335	291/321	59
**SF+5% PUE**	**1**	31	56	32	10
	**2**	278	335	293/319	59

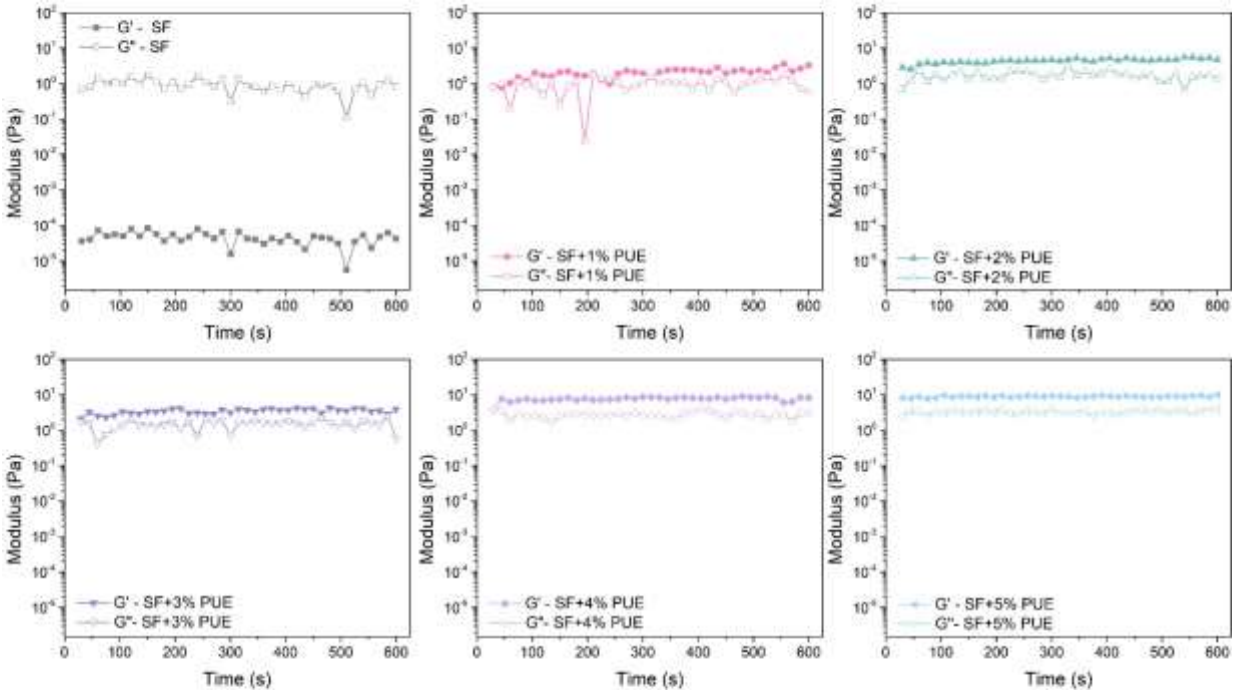

Fig. S3. Rheological time-sweep curves of SF and SF hydrogels containing different concentrations of PUE (1-5%).

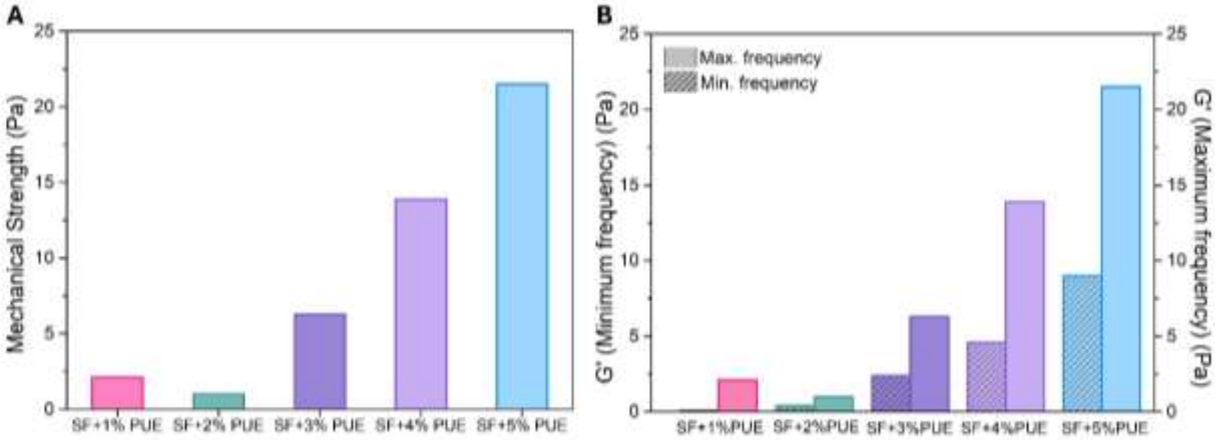

Fig. S4. Rheological analysis. **(A)** Mechanical strength as a function of puerarin (PUE) concentration in the hydrogel, and **(B)** storage modulus (G′) at maximum and minimum frequencies as a function of PUE concentration for silk fibroin (SF) solution and SF-based hydrogels containing different PUE concentrations (1-5%).
